# Effectiveness of a multi-component community-based care approach for older people at risk of care dependency - results of a prospective quasi-experimental study

**DOI:** 10.1186/s12877-022-02923-w

**Published:** 2022-04-20

**Authors:** Lena Hasemann, David Lampe, Thomas Nebling, Ulrich Thiem, Wolfgang von Renteln-Kruse, Wolfgang Greiner

**Affiliations:** 1 AG 5 - Department of Health Economics and Health Care Management, Bielefeld University, School of Public Health, Universitaetsstrasse 25, 33615 Bielefeld, Germany; 2 Department Care Management, Techniker Krankenkasse, Bramfelder Strasse 140, 22305 Hamburg, Germany; 3 Center for Geriatrics and Gerontology, Albertinen-Haus, Sellhopsweg 18-22, 22459 Hamburg, Germany; 4 University Medical Center Hamburg, Martinistrasse 52, 20251 Hamburg, Germany; 5 Emeritus University Medical Center Hamburg, Martinistrasse 52, 20251 Hamburg, Germany

**Keywords:** elderly, multi-component community-based care approach, intervention, functional decline, progression in long-term care grade, morbidity, health-related quality of life, effectiveness, evaluation, claims data

## Abstract

**Background:**

Due to demographic changes, the elderly population in western countries is constantly growing. As the risk of functional decline and multimorbidity increases with age, health care systems need to face the challenge of high demand for health care services and related costs. Therefore, innovative health care approaches and geriatric screenings are needed to provide individualised care. This study aims to expand the state of research by investigating the effectiveness of a multi-component care approach for the elderly in a German community setting.

**Methods:**

A prospective, quasi-experimental study was initiated by statutory health insurance (SHI) companies. The innovative care approach includes a geriatric assessment, a case and network management as well as digital supporting tools and was implemented at the Center for Geriatrics and Gerontology (Albertinen Haus, Hamburg-Eimsbuettel). Participants of the intervention were compared to matched controls recruited in comparable urban areas. The primary outcome measure was the progression in long-term care grade during the period of observation (21 months), which was analysed on the basis of SHI claims data. Secondary endpoints were morbidity, mortality and self-reported health-related quality of life (HRQoL) measured by SF-36.

**Results:**

Overall, 2,670 patients (intervention group (IG) n=873; control group (CG) n=1,797) were analysed. Logistic regression analysis showed no statistically significant difference in progression of long-term care grade between IG and CG (Odds Ratio (OR)=1.054; 95% confidence interval (CI) 0.856-1.296; p-value=0.616). Differentiated analyses indicated an initial effect, which might be attributable to the geriatric assessment. However, an adapted regression model resulted in a reversed but even non-significant effect (OR=0.945; 95% CI 0.757-1.177; p-value=0.619). While secondary analyses of long-term care grade, mortality and HRQoL did not show intervention effects, a statistically significant relative change of 0.865 (95% CI 0.780, 0.960; p-value=0.006) in morbidity indicated a potential benefit for the IG.

**Conclusions:**

The analyses did not reveal a significant effect of the community-based intervention on the primary outcome and thus we are not able to recommend a transfer into SHI standard care. Tendencies in secondary analyses need to be proved in further research.

**Trial registration:**

German Clinical Trials Register, retrospective registration on February 01, 2022 (DRKS00027866).

**Supplementary Information:**

The online version contains supplementary material available at 10.1186/s12877-022-02923-w.

## Background

Due to increasing life expectancy and low birth rates, all regions in Europe are experiencing aging of their populations [1]. According to population projections for Germany, demographic changes will result in an increase of the proportion of people aged ≥ 67 years from 19% in 2018 to 24%-30% in 2060 [2]. A common challenge in the aging population is functional decline, i.e. the ongoing impairment in different functional abilities like activities of daily living, mobility, cognition etc. Due to multimorbidity and age-associated changes in most physiological systems, e.g. sensory, central nervous or cardiovascular system, functional decline leads to loss of independence and an increased risk for adverse health outcomes [3]. Frailty, a geriatric syndrome characterised by functional decline, and multimorbidity lead to higher health care service utilisation and costs [4–6]. Thus, the elderly population is of particular importance for current and future Public Health.

As modern health care systems usually focus on single illnesses, the management of patients with complex health problems remains insufficient. To successfully face demographic trends, screening for functional impairment and frailty, case identification and tailored holistic management of frailty are required [7, 8]. Different concepts based on the principle of ‘reablement’ have been recognised as promising approaches for supporting older people’s physical activity and functional independence and ensuring a self-determined life [9–11]. However, evidence from Germany regarding the effectiveness of comprehensive interventions based on the reablement principle is lacking.

Against this backdrop, a new care approach (‘NetzWerk GesundAktiv’ - NWGA) was developed and evaluated with a focus on appropriateness for standard care in German statutory health insurance (SHI). The complex intervention incorporated a geriatric screening, a case management, community-based activities of prevention and health promotion as well as digital supporting tools (e.g. tablet, online platform). We aimed to evaluate the effectiveness of this multi-component community-based care approach for older people with functional impairments in terms of progression in need for care and self-reported health-related quality of life (HRQoL).

## Methods

### Study design

The prospective, quasi-experimental study was carried out in the region of Hamburg-Eimsbuettel. It was hypothesised that the multi-component intervention would prevent progression in care dependency. The project was initiated by SHI companies (Techniker Krankenkasse (TK), Barmer, DAK Gesundheit, Knappschaft) and was implemented in 2017 in cooperation with the Center for Geriatrics and Gerontology (Albertinen Haus, Hamburg-Eimsbuettel), Bielefeld University (School of Public Health, Department of Health Economics and Health Management), Johanniter-Unfall-Hilfe e. V. (Regional Association Hamburg), CIBEK technology + trading GmbH, NXI GmbH & Co. KG and VDI/VDE Innovation + Technik GmbH.

Candidates for the intervention group (IG) were contacted in the pilot region, while potential controls (CG) were recruited in other German urban areas, which were comparable to the pilot region in terms of socio-demographic and infrastructural aspects. Participants were eligible if they met the following inclusion criteria: Age ≥ 70, increased risk of loss of independence as measured by self-reported LUCAS functional ability index (LUCAS-FI was developed and validated in the ‘Longitudinal Urban Cohort Aging Study’ and classifies people without need for long-term care as ROBUST (3-6 resources and 0-2 risks), postROBUST (3-6 resources and risks), preFRAIL (0-2 resources and risks) or FRAIL (0-2 resources and 3-6 risks) [3]), long-term care grade 3 or less and written informed consent. To ensure target group specificity and to enable efficient use of health care resources, ‘increased risk of loss of independence’ was operationalised as LUCAS-FI stages postROBUST, preFRAIL and FRAIL. Exclusion criteria were not having fluent German language skills and living in a geriatric care facility.

A pre-analysis of TK claims data was conducted to identify the proportion of progression in long-term care grade among older adults in 2013 and 2014. The sample size calculation was based on the appraisal of experts, assuming a constant proportion of progression in long-term care grade within the IG and an increase of 22-23% in the CG. To improve the precision of estimations, IG to CG ratio was chosen to be 1:2 [12]. Given a power of 80%, a significance level of 5% and a dropout rate of 20%, the required sample size was n=1,000 (IG) and n=2,000 (CG) participants. After completion of the recruiting process, suitable CG participants were assigned to each IG participant via exact matching (1:2) by means of the following criteria: LUCAS-FI/long-term care grade, year of birth, gender, marital status, number of people living in the household.

### Intervention

The innovative care approach (NWGA) was inspired by the concept of reablement, which is described as *“[…] person-centred, holistic approach that aims to enhance an individual’s physical and/or other functioning, to increase or maintain their independence in meaningful activities of daily living at their place of residence and to reduce their need for long-term services”* *[*13*]*. The NWGA combined several elements of health and social care, involved different stakeholders and thus formed a community-based network to assist and support the elderly participants as well as their relatives. An initial geriatric assessment was conducted by the coordinating authority at the Center for Geriatrics and Gerontology to identify each IG participants’ needs and requirements. On the basis of the assessment, which comprised the examination of social aspects and health conditions as well as physical performance tests, experts generated an individual support plan by means of case conferences. Principles of the new care approach were ‘rehabilitation before and during long-term care’, ‘consultation, support, dementia’, ‘supporting relatives’, ‘health literacy’ and ‘people and technology’. Intervention services included e.g. basic and detailed case management consultations, expert-moderated group consultations and a network management to promote and mediate local offers in the field of health and exercise. Digital supporting tools for personal assistance were provided, continuously extended and improved. These tools allowed to consult involved parties by internet or video calls, included treatment and medication schedules, provided health information etc. Detailed information on the intervention can be found in Additional file 1: Table 1.

### Data collection and outcome measures

The analysis was conducted on the basis of primary and secondary data. To protect privacy and to ensure data protection, researchers received pseudonymised data without access to personal identifying information. Primary data were collected at baseline (T0) as well as after 12 (T1) and 21 months (T2). Self-reported measures included socio-demographic characteristics (T0), long-term care grade, functional ability index (LUCAS-FI) and HRQoL (T0-T2). The survey documents were provided to participants by their SHI company. Data entry was executed by Albertinen-Haus (IG T0) and Bielefeld University (CG T0, T1 and T2). Additionally, SHI companies provided pseudonymised claims data, covering the observation period as well as the year prior to inclusion.

The primary outcome was the progression in participants’ need for care, operationalised as a binary measurement of at least one request for an initial classification or an upgrade in long-term care grade. For better accuracy, the analysis was based on claims data. By January 2017 a new instrument for assessing the individual need of care was introduced to the German social long-term care insurance and replaced the previous long-term care levels [14]. It differentiates five long-term care grades which allow to classify the type and severity of the impairment [15]. Secondary outcomes were related to the grade of long-term care at the end of the period of observation, morbidity, mortality and the participants’ self-reported HRQoL. Morbidity status was evaluated with the updated Charlson Comorbidity Index (CCI) Score [16, 17] on the basis of main and secondary ICD 10-diagnoses documented in outpatient and inpatient settings. Eligible categories for CCI Score are 0 for healthy participants, 1-2 for mild, 3-4 for moderate and ≥ 5 for severe comorbidity [18]. HRQoL-data were collected by the widely-used SF-36v2 [19]. An additional table gives a detailed overview of all relevant parameters including their data source (Additional file 1: Table 2).

### Statistical analysis

Descriptive statistics were used to analyse the data. Baseline differences between IG and CG were examined using chi-square tests and t-tests. Relationships between dependent outcome variables and relevant predictors were investigated by regression analyses whereby the study group variable indicates the intervention effect. Logistic models (function glm(), package stats version 4.0.3 [20]) were applied to estimate the primary outcome (progression in long-term care grade) and mortality. The long-term care grade after 21 months represents an ordinal dependent variable and therefore requires a proportional odds model (function polr(), package MASS version 7.3-53 [21]). Moreover, an Ex-Gaussian distribution regression model formed the best fitting of CCI Score (function gamlss(), package gamlss version 5.3-2 [22]) and linear regression models were used to estimate HRQoL scores (function lm(), package stats version 4.0.3/ 4.0.4 [20]). An additional table gives an overview of outcomes, related regression models and estimators (Additional file 1: Table 3). Theoretical considerations informed the composition of the initial models. Potentially relevant predictor variables were age, gender, marital status, housing situation, study group, usage of a digital intervention component, the length of observation (death) as well as the previous year’s values for CCI Score, hospitalisations, days spent in hospital and outpatient consultations. Baseline functional status was documented by LUCAS-FI (participants without initial need for long-term care) or long-term care grade. As these aspects split the sample into two subgroups, they were combined in one predictor variable, including four categories (LUCAS-FI postROBUST (1), preFRAIL (2), FRAIL (3) and long-term care grade 1-3 (4)). In contrast to the CG, the majority of IG participants was recruited within the second half of 2018 which leads to the fact that their observation period (21 months) overlapped the COVID-19 pandemic. We addressed this by including the time point of enrolment as potential predictor variable in model selection process. The final regression term resulted from backward selection based on Akaike Information Criterion (AIC) [23], if necessary supplemented by study group variable. Bonferroni correction was used to consider multiple testing within the primary analysis. Due to the number of predictors included in regression analysis of progression in long-term care grade, the modified significance level was 0.56% (p*=0.05/9). For supplemental secondary analyses we applied a significance level of 5%. Whereas the primary outcome analysis is presented in the results, detailed model estimations of secondary outcomes are provided in additional tables (Additional file 1: Tables 4-15). Model fit and related reliability of the results were assessed by means of diagnostic plots (e.g. residual, qq and worm plot) (Additional file 1: Figs. 1-18). We assessed the discrimination of the fitted logistic regression models by receiver operating characteristic (ROC) curve and the area under the curve (AUC). Hosmer et al. [24] refer to an AUC of 50% to 70% as poor, 70% to 80% as acceptable, 80% to 90% as excellent and ≥90% as outstanding discrimination. The evaluation was performed using the open-source R Software [20].

## Results

**Fig. 1:**
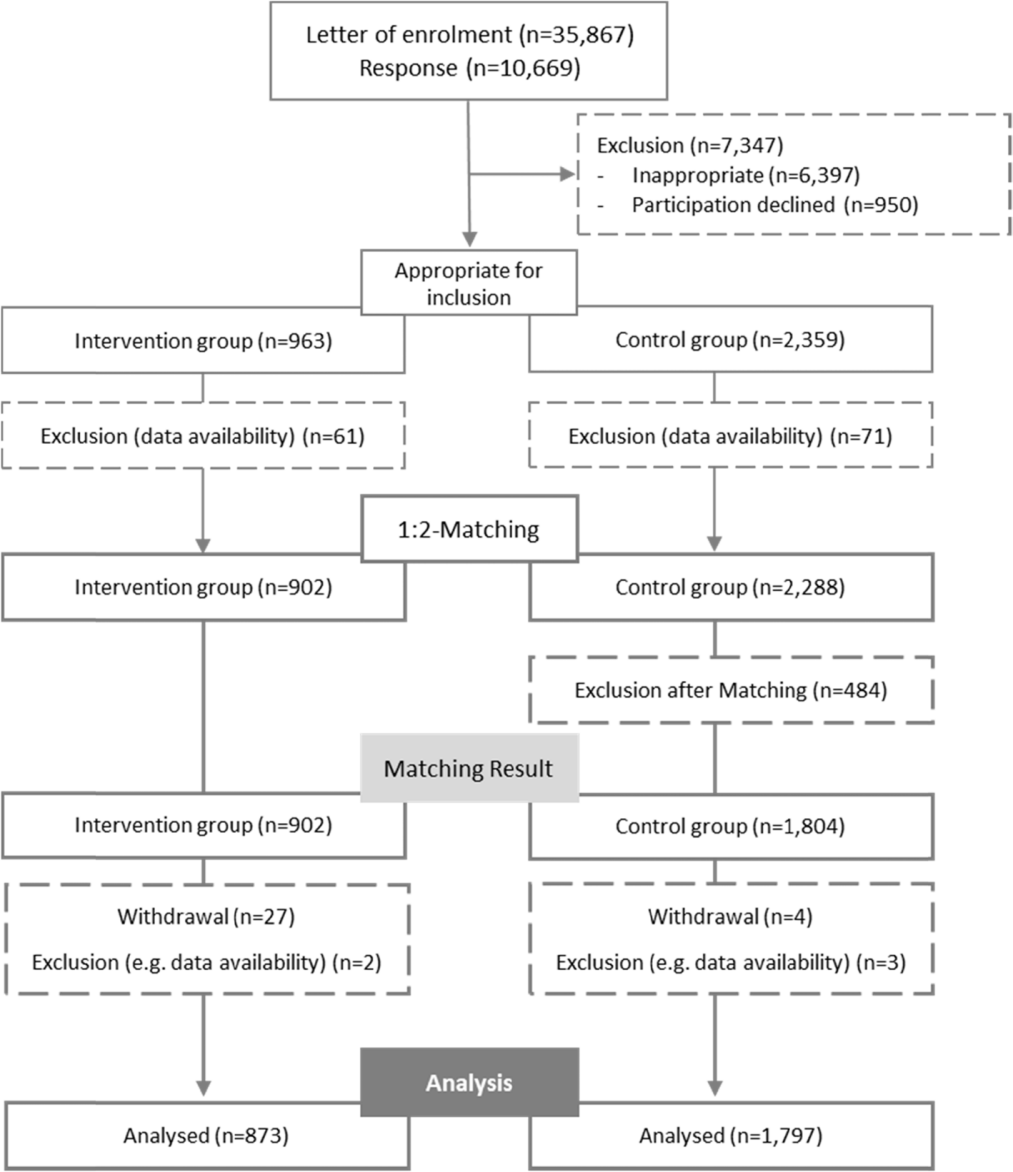
Flow chart of participants

The response rate to initial recruitment was about 30%. 6,397 potential participants were excluded due to defined inclusion and exclusion criteria, 950 declined to participate and additional 132 were excluded due to unavailable data. While the recruitment of the CG was completed in March 2018, IG recruitment was continued until December. Exact matching (1:2) involved 3,190 candidates and resulted in group sizes of 902 (IG) and 1,804 (CG). Dropout rates after matching in the IG (3.22%) and CG (0.39%) led to a final study population of 2,670 participants (Fig. 1). However, due to missing data or loss to follow-up, some analyses involved a reduced sample.

At baseline, the IG (n=873) and CG (n=1,797) did not differ significantly in socioeconomic characteristics or functional status (Table 1). The mean age of the mainly female (65.62%) study population was 80 years. About a half of both groups was married or in a long-term partnership and lived in a two-person household. Most participants’ functional status was classified as LUCAS-FI-stages frail (42.04% IG, 40.79% CG) and postROBUST (IG 36.88%, 37.90%, CG). Only 9.39% of IG and 8.85% of CG participants were formalised to long-term care grade (1-3) at enrolment. Analyses of the IG showed that participants utilised on average 18 intervention services and about 37% of them continuously used some form of digital supporting tool.

**Table 1:** Baseline characteristics

	Intervention (n=873)	Control (n=1,797)	p-value
**Age,** ø	80.13 (SD=5.35)	80.32 (SD=5.41)	0.3998
**Gender**			
female 0/1	591 (67.70%)	1,161 (64.61%)	0.1252
male 0/1	282 (32.30%)	636 (35.39%)	
**Marital status**			
married/long-term partnership	436 (49.94%)	933 (51.92%)	0.3589
unmarried/divorced/widowed	437 (50.06%)	864 (48.08%)	
**Housing situation**			
1 person	426 (48.80%)	845 (47.02%)	0.7885
2 people	443 (50.74%)	941 (52.37%)	
3 people	2 (0.23%)	7 (0.39%)	
≥ 4 people	2 (0.23%)	4 (0.22%)	
**LUCAS-FI/long-term care grade**			
postROBUST	322 (36.88%)	681 (37.90%)	0.8327
preFRAIL	102 (11.68%)	224 (12.47%)	
FRAIL	367 (42.04%)	733 (40.79%)	
long-term care grade 1-3	82 (9.39%)	159 (8.85%)	

*SD=Standard Deviation*

### Progression in long-term care grade

Compared to the year before enrolment (Table 2, A), progression in long-term care grade affected a higher proportion of the population in the first year of the study (14.43% IG, 11.35% CG) (B). Within the second year (up to 21 months of follow-up), a decrease of percentages of the IG population contrasted with a further increase in CG (C). This indicated an initial effect, which might have been attributable to the assessment, which was part of the intervention and might have led to recommendations regarding or an upgrade in long-term care grade. An adapted analysis, excluding the first 6 months, counteracted this potential effect and resulted in a similar level of about 18% of the IG and CG who experienced at least one progression in long-term care grade (E).

**Table 2:** Descriptive analysis of progression in long-term care grade (differentiated according to time periods A-E)

Progression in long-term care grade	Intervention (n=873)	Control (n=1,797)
A: Previous year	60 (6.87%)	119 (6.62%)
B: Study period - 1 to 12 months	126 (14.43%)	204 (11.35%)
C: Study period - 13 to 21 months	101 (11.57%)	232 (12.91%)
D: Study period - 1 to 21 months	209 (23.94%)	399 (22.20%)
E: Study period excl. assessment-effect - 7 to 21 months	163 (18.67%)	341 (18.98%)

A logistic model was used to estimate the progression in long-term care grade, considering the observation period of 21 months (Table 3). According to this analysis, the participants of the IG had a non-significant 1.054-fold chance (Odds Ratio (OR)=1.054; p=0.616) to experience a progression in long-term care grade compared to the CG. The most relevant predictors were baseline LUCAS-FI/long-term care grade. The evaluation of model discrimination resulted in an AUC of 74.1% (Additional file 1: Fig. 2) [24].

**Table 3:** Model estimation of progression in long-term care grade (Study period - 1 to 21 months)

	OR	95%-CI	p-value
Effect (IG vs. CG)	1.054	0.856, 1.296	0.616
Age	1.109	1.089, 1.130	<0.001**
LUCAS-FI preFRAIL (baseline)	2.226	1.570, 3.145	<0.001**
LUCAS-FI FRAIL (baseline)	3.285	2.570, 4.227	<0.001**
Long-term care grade 1-3 (baseline)	2.520	1.757, 3.606	<0.001**
CCI Score (previous year)	1.094	1.043, 1.147	<0.001**
Hospital visits (previous year)	1.120	1.019, 1.230	0.017*
Outpatient visits (previous year)	1.010	1.002, 1.018	0.009*
Length of observation	0.852	0.791, 0.914	<0.001**

*CI=Confidence Interval*

*statistical significance (p<0.05), ** statistical significance considering Bonferroni correction (p<0.0056)

The adapted model, which excluded a potential effect of the assessment, yielded a non-significant OR of 0.945 (p=0.619) for the study group (Table 4). However, statistically significant predictors were e.g. age, LUCAS-FI/long-term care grade at baseline and CCI Score calculated by means of the diagnoses of the previous year (Additional file 1: Table 4). Diagnostic procedures yielded an AUC of 73.1% (Additional file 1: Fig. 4).

**Table 4:** Model estimations (intervention effect) of progression in long-term care grade (excl. assessment-effect), long-term care grade, morbidity, mortality and HRQoL

	Effect (IG vs. CG)	95%-CI	p-value
**Progression in long-term care grade - excl. assessment-effect** (Study period - 7 to 21 months)	OR=0.945	0.757, 1.177	0.619
**Long-term care grade** (After 21 months)	OR=0.958	0.787, 1.163	0.665
**Morbidity (CCI Score)** (Study period - 1 to 21 months)	Exp(ß)=0.865	0.780, 0.960	0.006*
**Mortality** (Study period - 1 to 21 months)	*Unreliable estimation*		
	*OR<0.001*	*<0.001, <0.001*	<0.001*
**HRQoL (SF-36v2 scales)** (After 21 months)			
Physical functioning	ß=0.173	-2.328, 2.673	0.892
Physical role functioning	ß=0.204	-2.093, 2.501	0.862
Bodily pain	ß=0.133	-2.543, 2.809	0.922
General health perceptions	ß=0.721	-1.079, 2.522	0.432
Vitality	ß=0.731	-1.193, 2.656	0.456
Social role functioning	ß=0.726	-2.082, 3.534	0.612
Emotional role functioning	ß=1.451	-1.452, 4.355	0.327
Mental health	ß=-0.342	-4.732, 4.049	0.879

*OR= Odds Ratio; ß=Regression Coefficient*; *CI=Confidence Interval*

*statistical significance (p<0.05)

### Long-term care grade

**Fig. 2:**
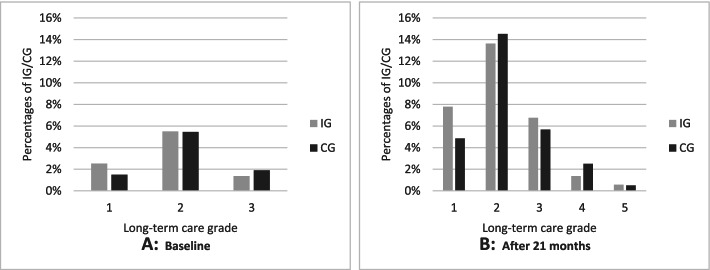
Descriptive analysis of long-term care grade at baseline (**A**) and after 21 months (**B**)

The percentage of participants in each study group who were in need for care increased from 9.39% (IG) and 8.85% (CG) at baseline to 30.13% (IG) and 28.05% (CG) at the end of the study, respectively. Differentiations of long-term care grades at baseline and after 21 months can be seen in Fig. 2. Grade 2 was most frequent at both time points in both groups. After 21 months of observation, more participants were formalised to grade 1 in the IG compared to the CG. Overall, there were no substantial differences within the distributions. Regression analysis yielded a similar result (Table 4). The proportional odds model revealed no significant effect of the intervention on long-term care grade after 21 months of observation (OR=0.958, p=0.665). Diagnostic plots showed small inaccuracies for higher values (Additional file 1: Figs. 5 and 6).

### Morbidity

**Fig. 3:**
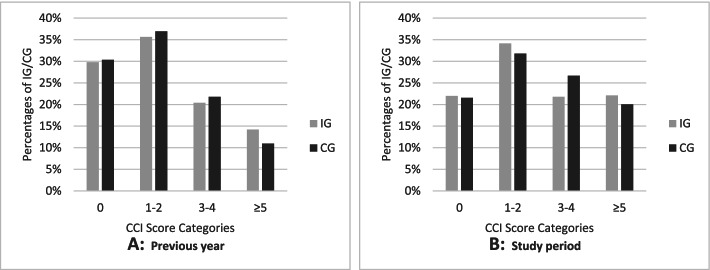
Descriptive analysis of CCI Score on the basis of documented diagnoses within the previous year (**A**) and the study period of 21 months (**B**)

Mean CCI Score on the basis of diagnoses from outpatient and inpatient treatment within the year before enrolment was 2.13 (SD=2.20) in IG and 2.03 (SD=2.06) in CG. The percentage of subjects who exhibited no relevant diagnosis for comorbidity calculation was about 30% in both groups (Fig. 3). Considering the study period, the percentage was 21.99% in IG and 21.53% in CG. Overall, participants tended to show higher scores within the study period compared to the previous year. On average, CCI Score based on diagnoses documented within the observation period was slightly lower in the IG (2.71, SD=2.49) than in the CG (2.77, SD=2.53). Regression analysis using Ex-Gaussian distribution confirmed a significantly lower (Exp(ß)=0.865, p=0.006) score of IG compared to CG participants (Table 4). Thus, the relative change in mean CCI Score is 0.865. Model diagnostics showed small deviations for lower values (Additional file 1: Figs. 7 and 9).

### Mortality

Within the observation period of 21 months, 4.12% of participants in the IG and 2.89% of participants in the CG population died. Due to the small number of cases, the analysis of mortality rates per month did not show clear peaks attributable to the first wave of COVID-19 pandemic in Germany. Likewise, the logistic regression model failed to provide reliable results (Table 4). Besides implausible coefficient estimates, the residual plot had uncovered definite patterns (Additional file 1: Fig. 10).

### Health-related quality of life

Compared to average values at baseline, both the IG and the CG showed a decrease of about 1 to 3 points on each SF-36v2-scale. The smallest changes were seen in bodily pain, which was 46.41 (SD=26.27) in IG and 46.47 (26.11) in CG participants at enrolment. IG participants had a slightly higher unadjusted mean score (2.57 points) in the mental health scale compared to CG participants after 21 months of observation (Table 5). However, as the threshold is set at 3 to 5 points [25, 26], no clinically relevant differences between IG and CG were found in unadjusted mean SF-36v2 scores. Linear regression analyses did not indicate significant intervention effects on HRQoL (Table 4). Study group coefficients, except from the mental health scale, designated non-significantly higher values for IG participants. Diagnostic plots showed deviations at bottom and top margins (Additional file 1: Figs. 11-18).

**Table 5:** Descriptive analysis of SF-36v2 scales at baseline (A) and after 21 months (B)

	A: Baseline		B: After 21 months	
HRQoL (SF-36v2 scales)	Intervention Ø (SD)	Control Ø (SD)	Intervention Ø (SD)	Control Ø (SD)
Physical functioning (n=2.539)	46.55 (23.61)	48.69 (24.64)	44.73 (26.24)	45.28 (26.62)
Physical role functioning (n=2.535)	42.99 (22.09)	44.19 (23.40)	41.35 (23.64)	41.51 (23.56)
Bodily pain (n=2.514)	46.41 (26.27)	46.47 (26.11)	45.93 (26.70)	45.53 (26.75)
General health perceptions (n=2.556)	51.25 (18.08)	49.96 (18.47)	49.30 (18.99)	47.99 (18.96)
Vitality (n=2.532)	48.94 (18.86)	49.45 (19.19)	46.69 (19.55)	46.48 (20.27)
Social role functioning (n=2.551)	68.04 (26.58)	67.80 (27.34)	63.40 (28.65)	63.25 (28.93)
Emotional role functioning (n=2.513)	63.18 (28.02)	63.40 (29.64)	59.66 (30.06)	58.46 (30.04)
Mental health (n=2.529)	66.96 (19.00)	66.04 (19.81)	65.59 (19.80)	62.93 (20.49)

*ø=mean value, SD=standard deviation*

*Minimal Clinically Important Difference (MCID): 3-5*

## Discussion

The analyses did not reveal considerable health effects of the community-based intervention as applied in this study. Examining the primary outcome, we found a comparable percentage of participants who experienced at least one progression in long-term care grade within the study period in both study groups. Regression analyses adjusting for potential confounders confirmed these findings. The diagnostic procedures corresponded to an acceptable discrimination of the model [24]. Differentiated descriptive analyses suggested an unintended effect of the initial assessment, which was a part of the intervention. However, this assumption could not be proven on basis of the available data.

Likewise, regression analysis of long-term care grade distribution and HRQoL values at 21 months after enrolment did not yield a significant result regarding the study group variable. The only minor indication of a benefit of the intervention arises from morbidity analysis, which yielded a significantly lower mean CCI Score in the IG compared to the CG. Diagnostic plots of long-term care grade, HRQoL and morbidity analyses verified acceptable estimations. However, the difference in CCI Score is of questionable clinical importance. Additionally, as the power calculation of the study was based on the primary outcome, the result has to be interpreted with caution. Due to a small number of cases, reliable results in terms of mortality rates were not achievable.

In this study, we aimed to evaluate a multi-component, community-based care approach for older people at risk of functional decline and long-term care dependency. The intervention is aligned to the concept of reablement which focusses the promotion of independence for the elderly [27] and usually combines different elements to a holistic, patient-centered care approach [11]. A common understanding of reablement is lacking [28] and comparability with existing studies is limited. However, complex health programs focusing on reablement have been shown to be effective compared to usual care regarding HRQoL [29]. Besides, there is little evidence suggesting that reablement has an impact on mortality [9]. Our results do not support these findings. However, they confirm the overall inconclusive body of evidence with regard to the effectiveness of interventions for older people at risk of functional decline [7]. Some studies designate integrated care [30] and case management [31] to be beneficial for frail or ‘at-risk’ patients. In contrast, other researchers do not designate these approaches to be preferable compared to usual care [32, 33], which is in line with the current findings.

Common challenges of intervention studies arise from short study periods, heterogeneous groups, the selection of appropriate intervention components [33–35], high complexity and problems regarding the implementation of intervention components [7]. Additionally, more high-quality studies are needed to contribute to more robust evidence [9]. With regard to the present results, it remains unclear whether they can be reduced to an ineffective care approach or should instead be attributed to conditions of data availability or the short period of observation.

The following strengths and weaknesses should be considered. Although the initially calculated required number of cases was not reached, this did not lead to limitations because the drop-out rate was low. In general, randomised controlled trials (RCT) generate the highest quality of evidence. Since an RCT could not be realised in the pilot region, a quasi-experimental design was chosen to compare participants of the multi-component care approach (IG) with participants of usual care (CG). Substantial effort was made to ensure comparability of the study groups, e.g. matching and regression analyses including potential confounder variables. Nevertheless, it should be noted, that results might have been affected by latent variables, which were not covered in the present data. With regard to the transferability (generalisability) of the results, it has to be mentioned, that the percentage of women (65.5%) is somewhat higher compared to the general population of the same age in Germany (57.5%) [36]. This might be caused by stronger health awareness and willingness to participate within the female population [37]. Moreover, the cohort was relatively healthy as initially about 91% had no long-term care grade. In general, a selection bias of health-conscious candidates cannot be excluded and might have affected the results. Considering the first wave of COVID-19 pandemic, the time point of enrolment was included as potential confounder in our analyses. However, the variable was excluded by backward selection and accordingly seems to have no relevant impact on the present outcomes.

The analyses were based on primary and secondary data, which provide a broad basis of information on participants’ socio-demographic characteristics, functional status, and HRQoL as well as health service utilisation and related diagnoses. As claims data are not collected for research purposes, they suffer from some limitations, e.g. diagnoses are only documented if patients consult a physician in an outpatient setting or receive inpatient treatment [38]. Further challenges arise from data quality as well as the process of data preparation. Particularly, previous year’s values of (progression in) long-term care grade are of restricted informative value due to limited data availability and potential inaccuracies caused by the new instrument for assessing the individual need of care. Besides these challenges, methodological limitations, i.e. recall bias and social desirability, might have influenced primary data quality.

A specific constraint in data analysis is related to the method of model selection by AIC. Although the algorithm avoids alpha error accumulation to a certain extent, potential bias can not be excluded, and thus significance could be overestimated. As regression models, except from morbidity analysis, did not result in significant results referring to the predictor of interest (study group), this is negligible. Despite this, Bonferroni correction was used to avoid bias due to multiple testing within the analysis of the primary outcome. Finally, the CCI Score should be interpreted with caution, since it is usually applied to predict mortality (in hospital settings) and is rarely intended to be an outcome measure. Additionally, score calculation is based on administrative diagnoses, which depend on the coding habits of health care providers in outpatient and inpatient settings.

## Conclusion

Overall, current findings on the innovative care approach do not allow to recommend a transfer into SHI standard care. Thus, further research is needed to examine the benefit of multi-component interventions for people at risk of functional decline and loss of independence and their potentials to improve care. Studies should pay attention to the selection of effective intervention components and the definition of measurable outcomes. Furthermore, study designs of high evidence levels are particularly desirable.

## Supplementary information

**Additional file 1.** Details on intervention components, relevant parameters and analyses

## Data Availability

The datasets generated and analysed during the current study are of particular sensibility and high need for protection. Due to project specific agreements of data protection, they are not publicly available. On reasonable request, possibilities of data access for external researchers have to be proved. Requests can be made to the corresponding author.

## Abbreviations

AIC: Akaike Information Criterion
CCI: Charlson Comorbidity Index
CG: control group
HRQoL: health-related quality of life
IG: intervention group
LUCAS-FI: LUCAS functional ability index
NWGA: NetzWerk GesundAktiv
OR: Odds Ratio
SF-36v2: Short Form (36) Health Survey Version 2
SHI: Statutory health insurance
TK: Techniker Krankenkasse
