# Supplementary material for: Effectiveness of a multi-component community-based care approach for older people at risk of care dependency - results of a prospective quasi-experimental study
Source: BMC Geriatr. 2022 Apr 20;22:348. doi: 10.1186/s12877-022-02923-w (PMC9022407; doi:10.1186/s12877-022-02923-w)
Supplement: Supplementary file 1 — Additional file 1. Details on intervention components, relevant parameters and analyses [file 12877_2022_2923_MOESM1_ESM.pdf]

Additional File 1: Details on intervention components, relevant parameters and analyses

1. Overview of intervention components

Table 1: Overview of intervention components including details and time expenditure

Intervention component	Details	Time expenditure (approx.)
**Assessment and case conference**	- Interdisciplinary assessment of individual resources and risks, including:<br>- Medical conditions (e.g. medical anamnesis and examination, assessment of pain, mental health condition, depression status, frailty, nutrition, risk of falling)<br>- Social aspects (e.g. Housing situation, life orientation satisfaction, social environment, Instrumental Activities of Daily Living (IADL), care dependency)<br>- Physical performance (e.g. gait analysis, mobility test, assessment of strength and balance in extremities, cognition, Bioelectric impedance analysis)<br>- Development of an individual support plan based on the assessment results by experts in case conferences	4 h 45 min.
**Case management: Basic consultation**	- Communication with participants and service providers<br>- Coordination of (health) care utilisation recommended in the support plan<br>- Provision of information on various issues (e.g. prevention, health promotion, social care)	35 min.
**Case management: Detailed consultation**	- Provision of information and support in relation to social services and care services, financial aspects<br>- Adjustment of individual support plans if the participant's health status changes over time	1 h 30 min.
**Case management: Home visits**	- Home visits by case managers to provide advice and support for participants with limitations in their mobility	2 h 30 min.
**Case management: Group consultations**	- Expert moderated group consultations based on the idea of self-help groups<br>- Possibility to discuss specific health issues<br>- Promotion of social participation	1 h 30 min.
**Case management: Dementia consultation**	- Provision of information and support for participants at risk for dementia or who care for an affected person	45 min.
**Network management**	- Communication on and provision of an overview (event calendar/ website) of quality-assured and currently available offerings (health/exercise/sport/social) in the district<br>- Promotion of social participation, mobility and health-related quality of life of the participants	45 – 120 min.
**Digital supporting tool ('PAUL')**	- Digital tool for accessing the various support services and to interact with case managers or relatives<br>- Presentation of current local social and cultural events<br>- Café PAUL: senior-friendly courses/seminars on media/digital competencies	3 h per week (Café PAUL)

2. Overview of relevant parameters

Table 2: Overview of relevant parameters their functioning for the evaluation and their data source

Parameter	Matching variable	Outcome	Data source
Age	x		P+S
Gender	x		P+S
Marital status	x		P
Housing situation	x		P
LUCAS-FI	x		P
Long-term care grade	x	x	P+S
Progression in long-term care grade		x	S
Morbidity (CCI Score)		x	S
Mortality		x	S
HRQoL		x	P
P=Primary data, S=Secondary data			

3. Overview of outcomes and regression models

Table 3: Overview of outcomes, applied regression models, estimators and interpretations

Outcome	Model	R function	Estimator
Progression in long-term care grade (21 months)	Logistic regression model	glm(), package stats version 4.0.3 [20]	OR
Progression in long-term care - excl. assessment-effect (7 to 21 months)	Logistic regression model	glm(), package stats version 4.0.3 [20]	OR
Long-term care grade	Proportional odds model	polr(), package MASS version 7.3-53 [21]	OR
Morbidity (CCI Score)	Ex-gaussian distribution regression model	gamlss(), package gamlss version 5.3-2 [22]	Exp(ß)
Mortality	Logistic regression model	glm(), package stats version 4.0.3 [20]	OR
HRQoL	Linear regression model	lm(), package stats version 4.0.3 bzw. 4.0.4 [15]	ß
OR=Odds Ratio, ß=Regression Coefficient			

4. Detailed model estimations

4.1. Progression in long-term care grade – excl. assessment-effect

Table 4: Detailed model estimation of progression in long-term care grade – excl. assessment-effect (Study period - 7 to 21 months)

	**OR**	**95 %-CI**	**p-value**
Study group (IG vs. CG)	0.945	0.757; 1.177	0.619
Age	1.115	1.094; 1.138	**<0.001***
LUCAS-FI preFRAIL (baseline)	2.060	1. 424; 2.962	**<0.001***
LUCAS-FI FRAIL (baseline)	2.807	2.166; 3.664	**<0.001***
Long-term care grade I-III (baseline)	1.269	0.607; 2.475	0.503
Progression in long-term care grade (previous year)	2.063	1.015; 4.454	0.053
CCI Score (previous year)	1.096	1.043; 1.151	**<0.001***
Outpatient visits (previous year)	1.010	1.002; 1.017	**0.018***
Length of observation	0.924	0.862; 0.994	**0.029***
CI=Confidence Interval<br>**statistical significance (p<0.05)*			

4.2. Long-term care grade

Table 5: Detailed model estimation of long-term care grade (After 21 months)

	**OR**	**95 %-CI**	**p-value**
Effect (IG vs. CG)	0.958	0.787, 1.163	0.665
Age	1.101	1.082, 1.120	**<0.001***
LUCAS-FI preFRAIL (baseline)	2.539	1.796, 3.575	**<0.001***
LUCAS-FI FRAIL (baseline)	3.398	2.666, 4.361	**<0.001***
Long-term care grade I-III (baseline)	33.763	24.511, 46.820	**<0.001***
CCI Score (previous year)	1.086	1.039, 1.136	**<0.001***
Hospital visits (previous year)	1.124	1.026, 1.228	**0.011***
Outpatient visits (previous year)	1.008	1.000, 1.015	**0.028***
Length of observation	0.783	0.730, 0.839	**<0.001***
CI=Confidence Interval<br>**statistical significance (p<0.05)*			

4.3. Morbidity (CCI Score)

Table 6: Detailed model estimation of morbidity (CCI Score) (Study period - 21 months)

	**Exp(ß)**	**95 %-CI**	**p-value**
Effect (IG vs. CG)	0.865	0.780, 0.960	**0.006***
Age	1.011	1.001, 1.020	**0.026***
Gender (female)	0.813	0.733, 0.903	**<0.001***
LUCAS-FI preFRAIL (baseline)	0.938	0.800, 1.099	0.426
LUCAS-FI FRAIL (baseline)	1.166	1.044, 1.303	**0.007***
Long-term care grade I-III (baseline)	1.160	0.956, 1.406	0.133
CCI Score (previous year)	2.152	2.090, 2.216	**<0.001***
Hospital visits (previous year)	0.935	0.886, 0.988	**0.016***
Outpatient visits (previous year)	1.007	1.003, 1.011	**0.001***
Length of observation	0.831	0.794, 0.870	**<0.001***
CI=Confidence Interval<br>**statistical significance (p<0.05)*			

4.4. Mortality

Table 7: Detailed model estimation of mortality (Study period - 21 months)

	**OR**	**95 %-CI**	**p-value**
Effect (IG vs. CG)	<0.001	<0.001, <0.001	**<0.001***
Age	1.113	1.064, 1.166	**<0.001***
Gender (female)	0.632	0.376, 1.067	0.083
CCI Score (previous year)	1.112	0.993, 1.242	0.061
Hospital visits (previous year)	1.324	1.060, 1.617	**0.009***
Length of observation	0.388	0.331, 0.446	**<0.001***
CI=Confidence Interval<br>**statistical significance (p<0.05)*<br>*Unreliable estimation*			

4.5. Health-related quality of Life (HRQoL)

4.5.1. SF.PF - Physical functioning

Table 8: Detailed model estimation of 'Physical functioning' (After 21 months)

	ß	95 %-CI	p-value
Intercept	19.124	14.588, 23.66	**<0.001***
Effect (IG vs. CG)	0.173	-2.328, 2.673	0.892
Gender (female)	-1.825	-4.21, 0.561	0.134
LUCAS-FI preFRAIL (baseline)	-2.354	-6.143, 1.435	0.223
LUCAS-FI FRAIL (baseline)	-3.510	-6.288, -0.731	**0.013***
Long-term care grade I-III (baseline)	-5.108	-10.268, 0.052	0.052
Hospital visits (previous year)	1.419	-0.121, 2.958	0.071
Hospital length of stay (previous year)	-0.132	-0.273, 0.008	0.064
SF.PF (baseline)	0.357	0.293, 0.421	**<0.001***
SF.PF (T1)	0.237	0.181, 0.294	**<0.001***
corr. R^2: 0.284<br>*CI=Confidence Interval*<br>**statistical significance (p<0.05)*			

4.5.2. SF.RP - Physical role functioning

Table 9: Detailed model estimation of 'Physical role functioning' (After 21 months)

	ß	95 %-CI	p-value
Intercept	33.823	17.053, 50.592	**<0.001***
Effect (IG vs. CG)	0.204	-2.093, 2.501	0.862
Age	-0.185	-0.39, 0.02	0.077
LUCAS-FI preFRAIL (baseline)	-2.346	-5.869, 1.176	0.192
LUCAS-FI FRAIL (baseline)	-3.888	-6.385, -1.39	**0.002***
Long-term care grade I-III (baseline)	-6.491	-11.26, -1.722	**0.008***
Hospital visits (previous year)	1.348	0.298, 2.398	**0.012***
SF.RP (baseline)	0.312	0.254, 0.37	**<0.001***
SF.RP (T1)	0.232	0.179, 0.285	**<0.001***
corr. R^2: 0.2493<br>*CI=Confidence Interval*<br>**statistical significance (p<0.05)*			

4.5.3. SF.BP - Bodily pain

Table 10: Detailed model estimation of 'Bodily pain' (After 21 months)

	**ß**	**95 %-CI**	**p-value**
Intercept	44.366	25.217; 63.514	**<0.001***
Effect (IG vs. CG)	0.133	-2.543; 2.809	0.922
Age	-0.211	-0.447; 0.026	0.081
Gender (female)	-2.422	-4.959; 0.116	0.061
LUCAS-FI preFRAIL (baseline)	-3.125	-7.147; 0.897	0.128
LUCAS-FI FRAIL (baseline)	-2.181	-4.969; 0.608	0.125
Long-term care grade I-III (baseline)	-7.259	-12.362; -2.157	**0.005***
Outpatient visits (previous year)	-0.121	-0.213; -0.028	**0.011***
SF.BP (baseline)	0.284	0.226; 0.342	**<0.001***
SF.BP (T1)	0.238	0.183; 0.293	**<0.001***
corr. R^2*: 0.235*<br>*CI=Confidence Interval*<br>**statistical significance (p<0.05)*			

4.5.4. SF.GH - General health perceptions

Table 11: Detailed model estimation of 'General health perceptions' (After 21 months)

	**ß**	**95 %-CI**	**p-value**
Intercept	23.360	20.098, 26.622	**<0.001***
Effect (IG vs. CG)	0.721	-1.079, 2.522	0.432
LUCAS-FI preFRAIL (baseline)	-2.545	-5.29, 0.2	0.069
LUCAS-FI FRAIL (baseline)	-3.275	-5.154, -1.395	**0.001***
Long-term care grade I-III (baseline)	-5.969	-9.56, -2.379	**0.001***
SF.GH (baseline)	0.306	0.252, 0.36	**<0.001***
SF.GH (T1)	0.227	0.176, 0.279	**<0.001***
corr. R^2*: 0.2497*<br>*CI=Confidence Interval*<br>**statistical significance (p<0.05)*			

4.5.5. SF.VT - Vitality

Table 12: Detailed model estimation of 'Vitality' (After 21 months)

	**ß**	**95 %-CI**	**p-value**
Intercept	34.402	20.292, 48.511	**<0.001***
Effect (IG vs. CG)	0.731	-1.193, 2.656	0.456
Age	-0.147	-0.32, 0.026	0.096
Gender (female)	-1.465	-3.307, 0.377	0.119
LUCAS-FI preFRAIL (baseline)	-1.440	-4.35, 1.471	0.332
LUCAS-FI FRAIL (baseline)	-3.305	-5.335, -1.275	**0.001***
Long-term care grade I-III (baseline)	-6.605	-10.482, -2.729	**0.001***
SF.VT (baseline)	0.305	0.245, 0.365	**<0.001***
SF.VT (T1)	0.239	0.183, 0.295	**<0.001***

corr. R^2: 0.2554
CI=Confidence Interval
**statistical significance (p<0.05)*

4.5.6. SF.SF - Social role functioning

Table 13: Detailed model estimation of 'Social role functioning' (After 21 months)

	**ß**	**95 %-CI**	**p-value**
Intercept	53.196	32.846, 73.545	**<0.001***
Effect (IG vs. CG)	0.726	-2.082, 3.534	0.612
Age	-0.281	-0.534, -0.028	**0.030***
LUCAS-FI preFRAIL (baseline)	-2.455	-6.733, 1.824	0.261
LUCAS-FI FRAIL (baseline)	-4.731	-7.7, -1.763	**0.002***
Long-term care grade I-III (baseline)	-8.730	-14.31, -3.15	**0.002***
SF.SF (baseline)	0.277	0.218, 0.337	**<0.001***
SF.SF (T1)	0.244	0.188, 0.299	**<0.001***

corr. R^2: 0.2169
CI=Confidence Interval
**statistical significance (p<0.05)*

4.5.7. SF.RE - Emotional role functioning

Table 14: Detailed model estimation of 'Emotional role functioning' (After 21 months)

	ß	95 %-CI	p-value
Intercept	27.732	22.169, 33.294	**<0.001***
Effect (IG vs. CG)	1.451	-1.452, 4.355	0.327
Gender (female)	-2.189	-4.935, 0.557	0.118
LUCAS-FI preFRAIL (baseline)	-2.707	-7.151, 1.738	0.232
LUCAS-FI FRAIL (baseline)	-5.415	-8.385, -2.445	**<0.001***
Long-term care grade I-III (baseline)	-3.443	-9.289, 2.402	0.248
Hospital length of stay (previous year)	0.121	-0.015, 0.257	0.080
Outpatient visits (previous year)	-0.116	-0.218, -0.014	**0.026***
SF.RE (baseline)	0.306	0.251, 0.36	**<0.001***
SF.RE (T1)	0.287	0.235, 0.339	**<0.001***
corr. R^2: 0.276<br>*CI=Confidence Interval*<br>*statistical significance (p<0.05)*			

4.5.8. SF.MH - Mental health

Table 15: Detailed model estimation of 'Mental health' (After 21 months)

	ß	95 %-CI	p-value
Intercept	2.017	-11.832, 15.866	0.775
Effect (IG vs. CG)	-0.342	-4.732, 4.049	0.879
LUCAS-FI preFRAIL (baseline)	-1.642	-4.502, 1.218	0.260
LUCAS-FI FRAIL (baseline)	-2.904	-4.831, -0.977	**0.003***
Long-term care grade I-III (baseline)	-5.862	-9.564, -2.161	**0.002***
PAUL	2.495	-0.722, 5.713	0.129
Time point of enrolment2	20.854	7.3, 34.407	**0.003***
Time point of enrolment3	21.068	7.541, 34.596	**0.002***
Time point of enrolment4	20.781	6.16, 35.402	**0.005***
Time point of enrolment5	23.520	9.209, 37.832	**0.001***
Time point of enrolment6	22.131	7.718, 36.543	**0.003***
SF.MH (baseline)	0.336	0.28, 0.393	**<0.001***
SF.MH (T1)	0.293	0.239, 0.348	**<0.001***
corr. R^2: 0.3246<br>*CI=Confidence Interval*<br>*statistical significance (p<0.05)*			

5. Diagnostic plots

5.1. Progression in long-term care grade (21 months)

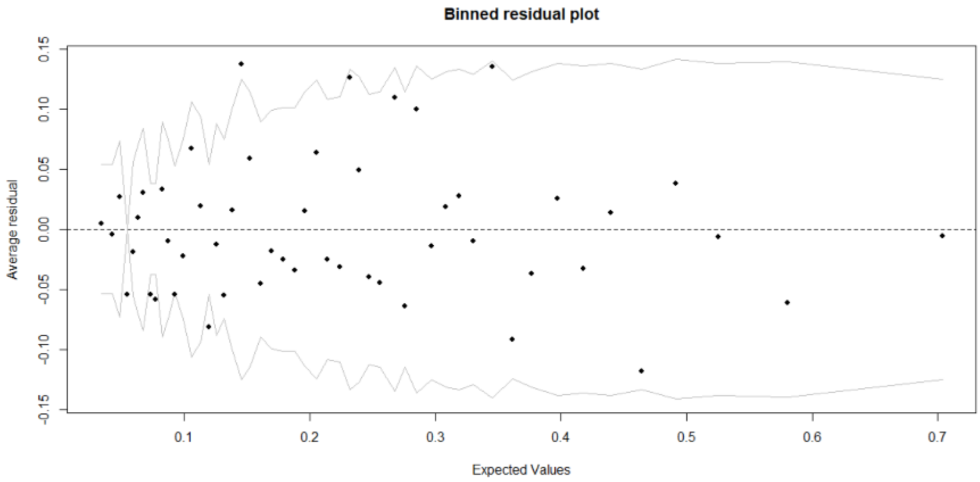

Figure 1: Binned residual plot for model estimation of progression in long-term care grade

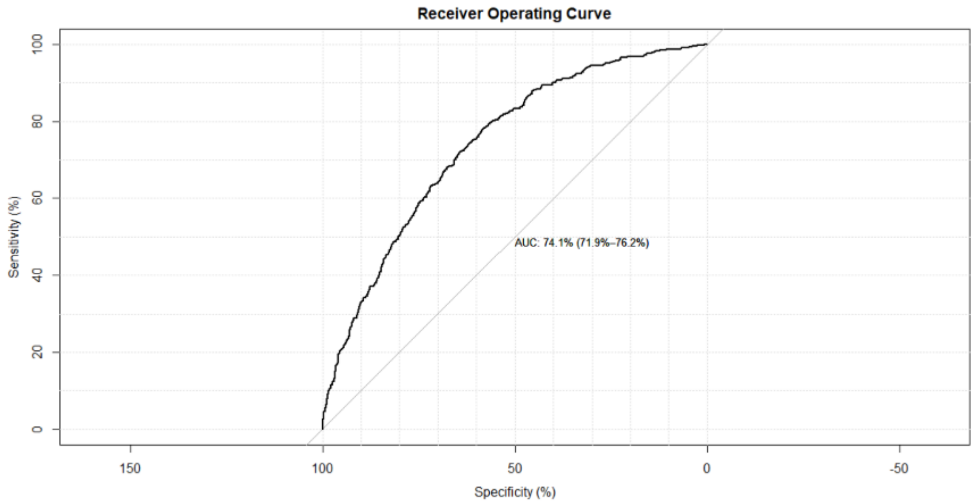

Figure 2: Receiver operating curve for model estimation of progression in long-term care grade

5.2. Progression in long-term care – excl. assessment-effect (7 to 21 months)

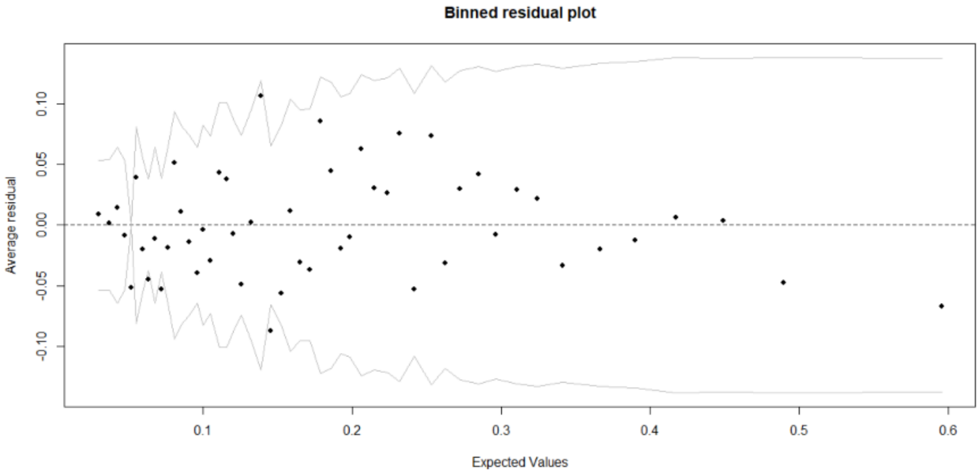

Figure 3: Binned residual for model estimation of progression in long-term care grade care – excl. assessment-effect

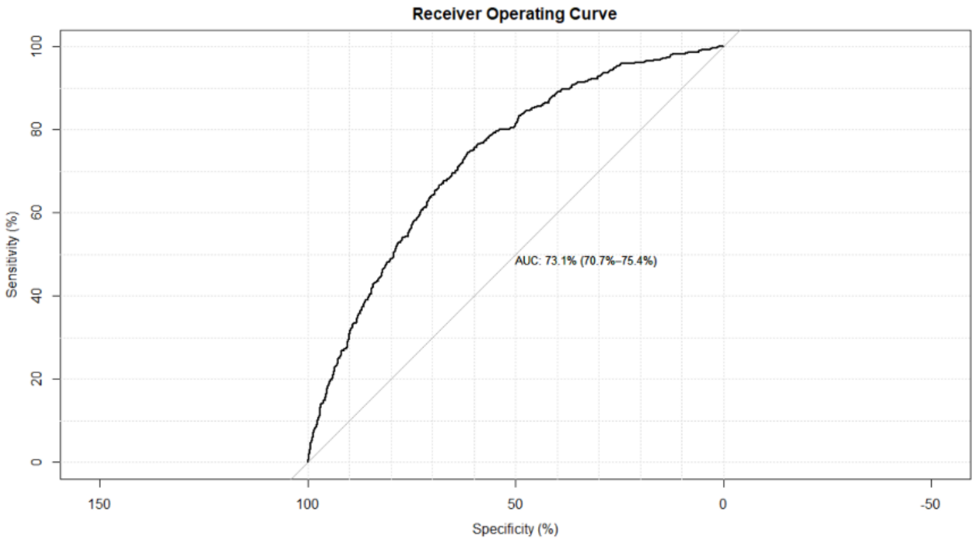

Figure 4: Receiver operating curve for model estimation of progression in long-term care grade care – excl. assessment-effect

5.3. Long-term care grade

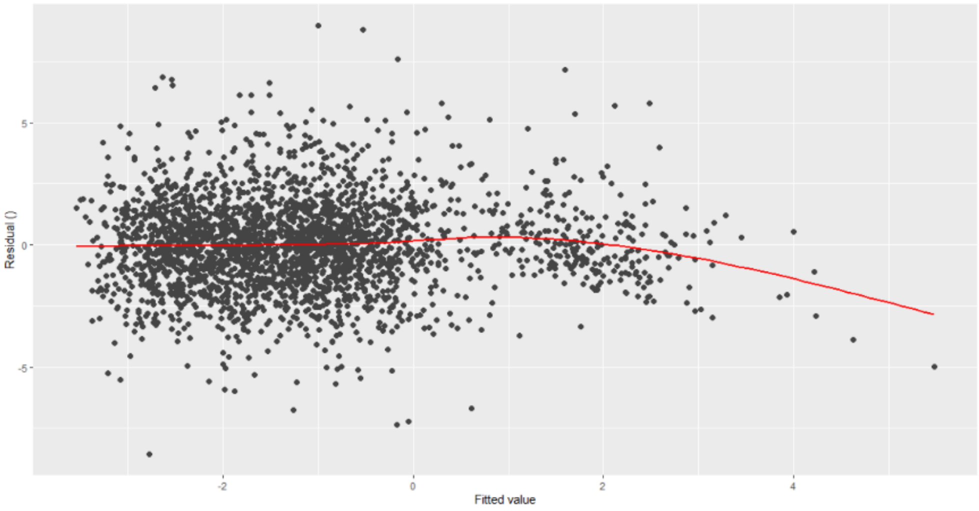

Figure 5: Residual plot for model estimation of long-term care grade

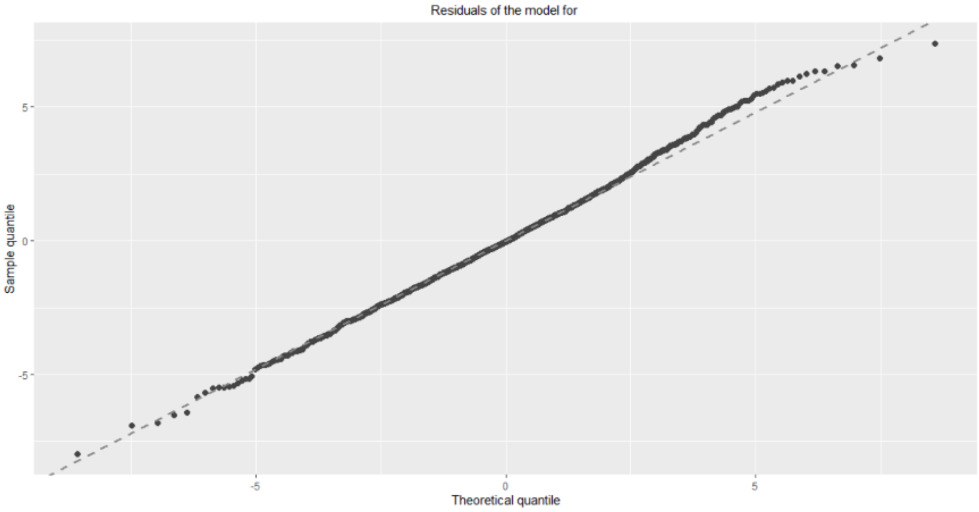

Figure 6: QQ-Plot for model estimation of long-term care grade

5.4. Morbidity (CCI Score)

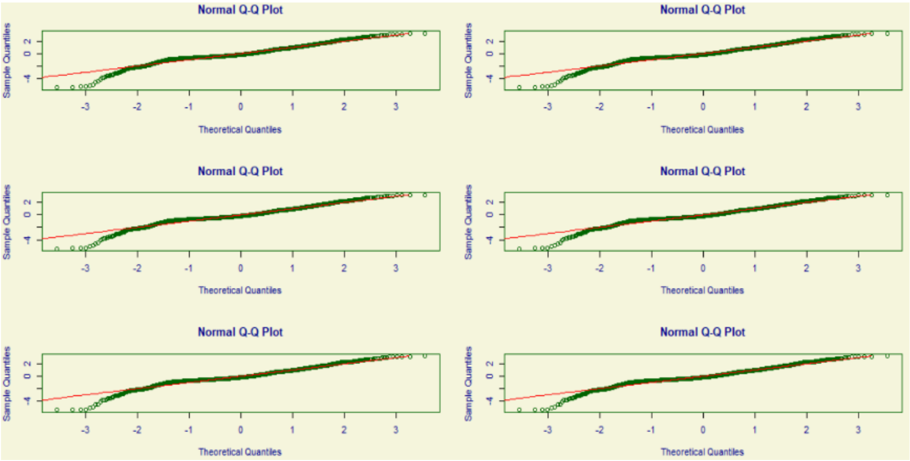

Figure 7: QQ-Plot for model estimation of morbidity

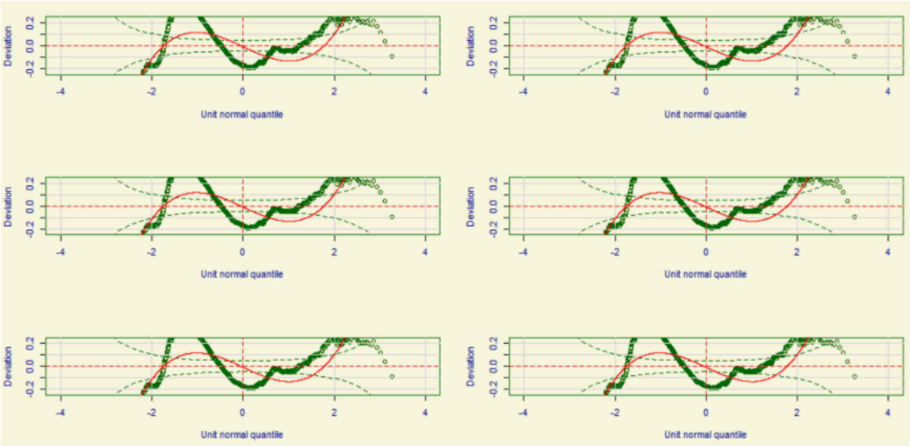

Figure 8: Worm-plot for model estimation of morbidity

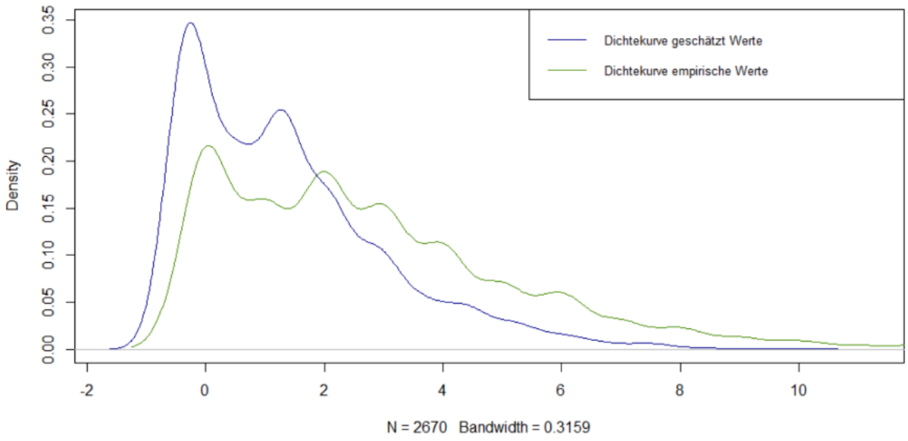

Figure 9: Density-plot for model estimation of morbidity

5.5. Mortality

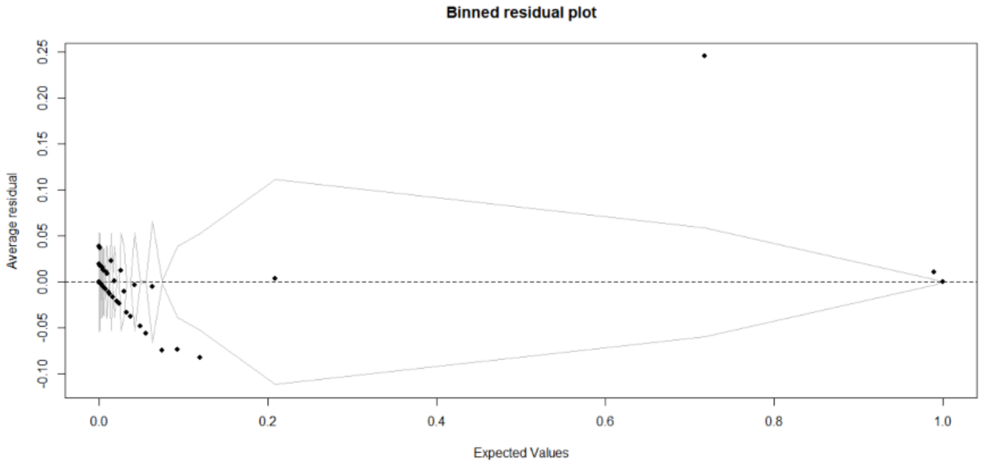

Figure 10: Binned residual plot for model estimation of mortality

5.6. Health-related quality of Life (HRQoL)

5.6.1. Physical functioning

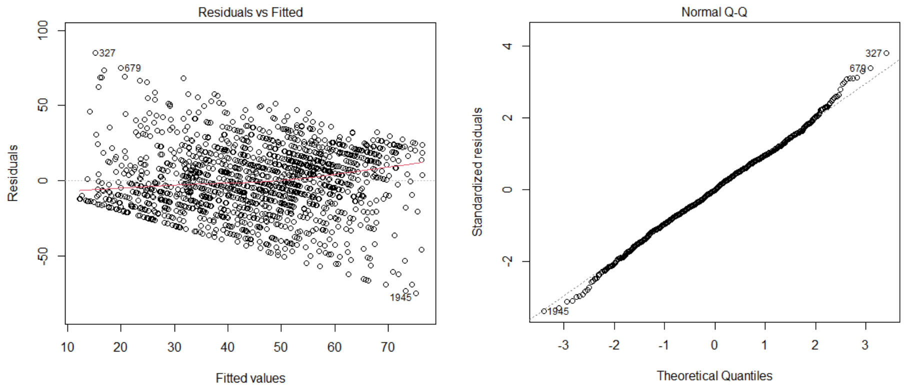

Figure 11: Residual and QQ plot for model estimation of 'Physical functioning'

5.6.2. Physical role functioning

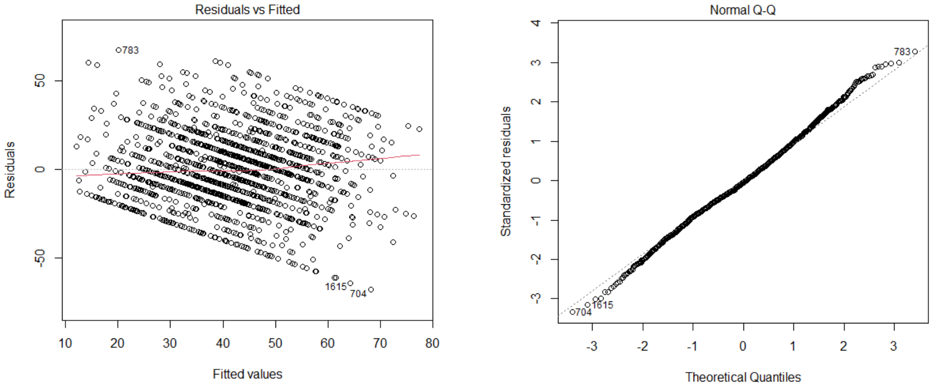

Figure 12: Residual and QQ plot for model estimation of 'Physical role functioning'

5.6.3. Bodily pain

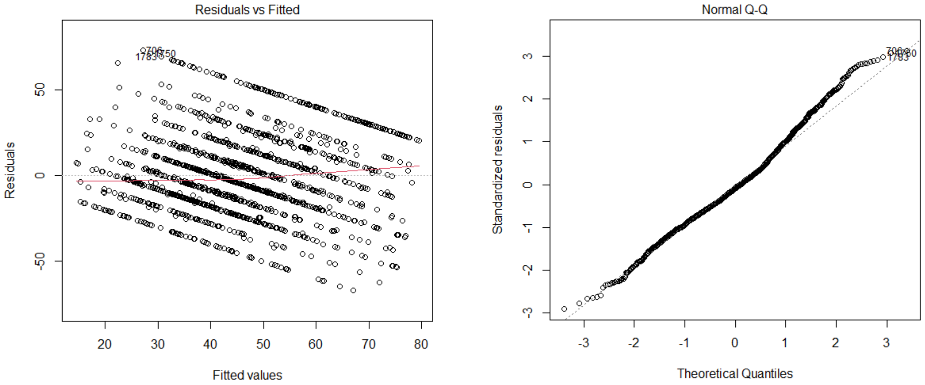

Figure 13: Residual and QQ plot for model estimation of 'Bodily Pain'

5.6.4. General health perceptions

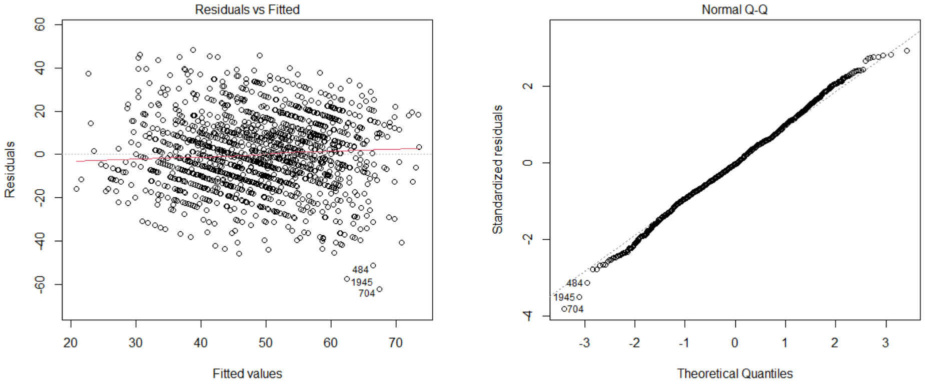

Figure 14: Residual and QQ plot for model estimation of 'General health perceptions'

5.6.5. Vitality

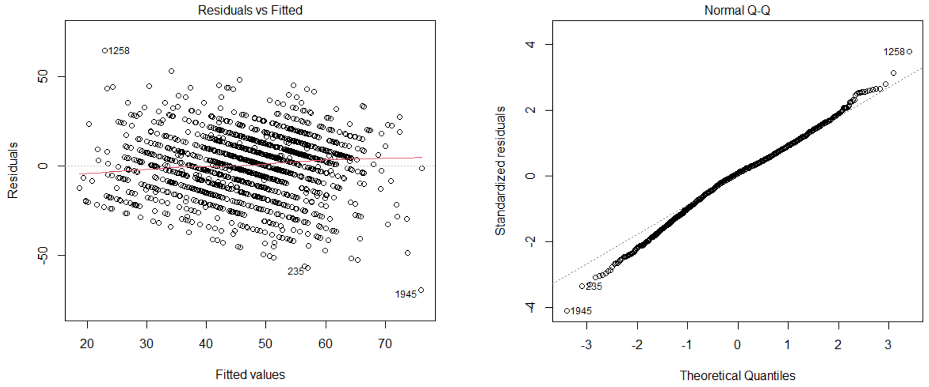

Figure 15: Residual and QQ plot for model estimation of 'Vitality'

5.6.6. Social role functioning

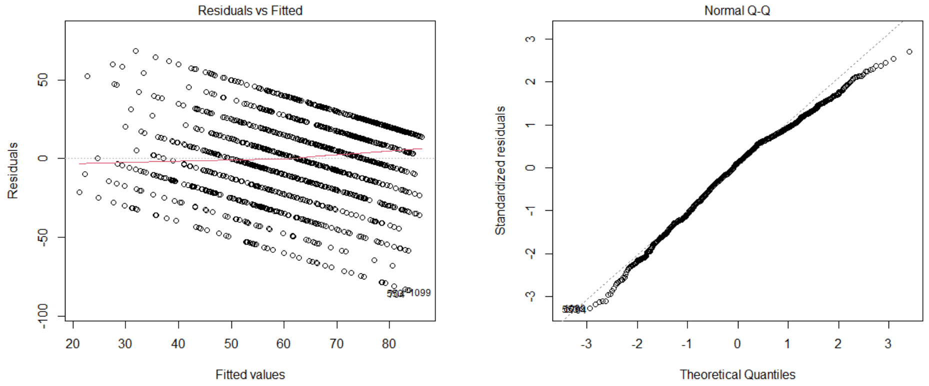

Figure 16: Residual and QQ plot for model estimation of 'Social role functioning'

5.6.7. Emotional role functioning

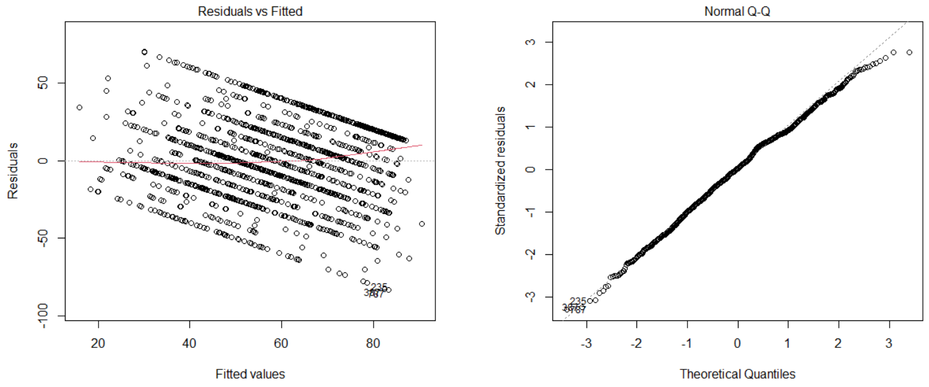

Figure 17: Residual and QQ plot for model estimation of 'Emotional role functioning'

5.6.8. Mental health

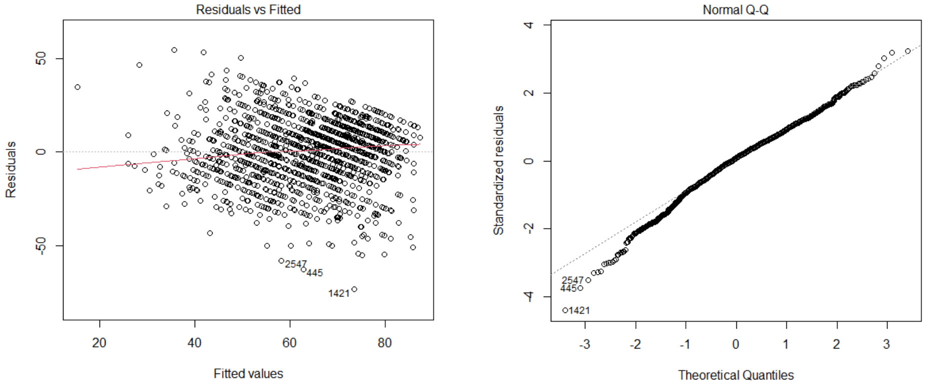

Figure 18: Residual and QQ plot for model estimation of 'Mental health'
